# Supplementary material for: Seasonal Paspalum vaginatum Physiological Characteristics Change with Agricultural Byproduct Biochar in Sandy Potting Soil
Source: Biology (Basel). 2022 Apr 7;11(4):560. doi: 10.3390/biology11040560 (PMC9025672; doi:10.3390/biology11040560)
Supplement: Supplementary file 1 [file biology-11-00560-s001.zip › biology-1613590-supplementary.pdf]

Supplementary Materials:

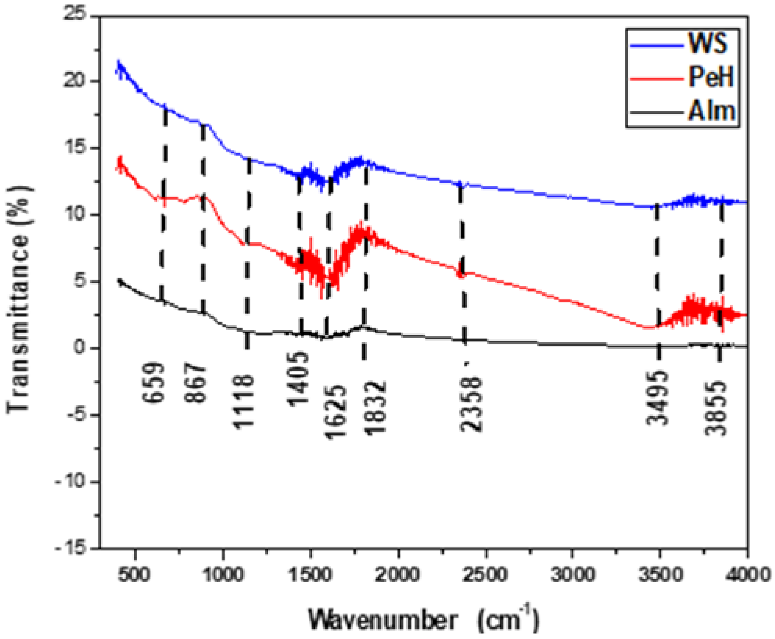

Figure S1. FTIR spectral analysis of three biochars made from peanut hulls, walnut shells, and almond shells.

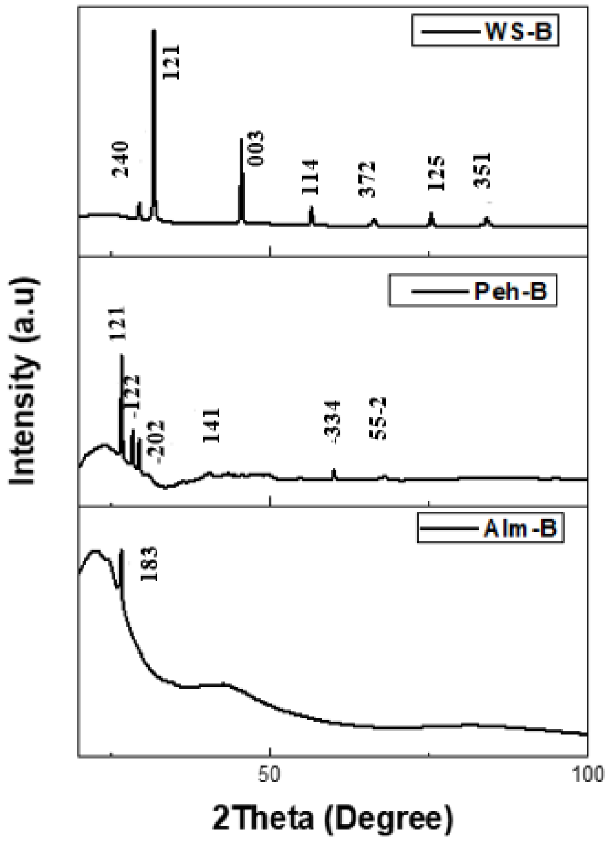

Figure S2. The XRD spectra of peanut hull biochar (Peh-B), walnut shell biochar (WS-B), and almond shell biochar (Alm-B).

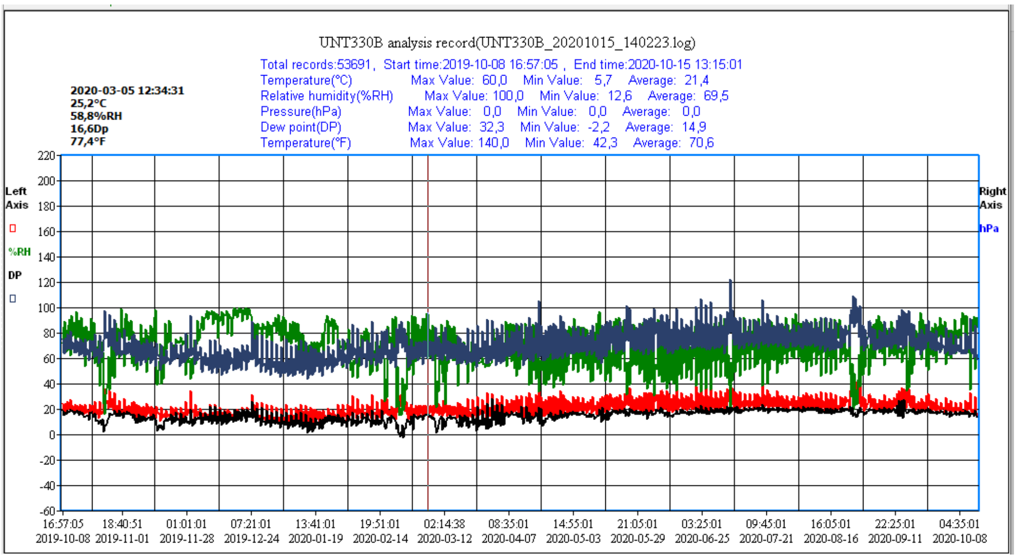

Figure S3. Figure depicting the temperature and humidity of *P. vaginatum* in pot and field conditions over a one-year period, as measured with a data logger UNI-T UT330A.
